# Periweaning failure to thrive syndrome (PFTS): A growing concern in swine health

**DOI:** 10.1186/s40813-025-00434-9

**Published:** 2025-04-10

**Authors:** Macarena Rodríguez-Ruiz, Librado Carrasco, Inés Ruedas-Torres, José M. Sánchez-Carvajal, Karola Fristiková, Carmen Álvarez-Delgado, Irene M. Rodríguez-Gómez, Jaime Gómez-Laguna, Francisco J. Pallarés

**Affiliations:** 1 Department of Anatomy and Comparative Pathology and Toxicology, Pathology and Immunology Group (UCO-PIG), International Excellence Agrifood Campus ’CeiA3’, UIC Zoonosis y Enfermedades Emergentes (ENZOEM), University of Córdoba, Córdoba, 14014 Spain; 2 Pathology Group, United Kingdom Health Security Agency (UKHSA), Porton Down, Salisbury, SP4 0JG Wiltshire UK

**Keywords:** Pig, PFTS, Failure to thrive, Periweaning, Growth retardation, Thymic atrophy

## Abstract

**Background:**

Porcine Periweaning Failure to Thrive syndrome (PFTS) is a complex and scarcely investigated syndrome that has been of increasing concern in the swine industry during the last decade. Its aetiology is believed to be multifactorial, and although both infectious and non-infectious factors may be involved, including a possible genetic predisposition, consistent association needs to be elucidated.

**Main Body:**

PFTS is characterised by growth retardation and non-specific clinical symptoms that may include progressive debilitation of weaned pigs that typically emerge within two to three weeks after weaning and repetitive oral behaviour such as chomping and licking. Currently, the diagnosis of the syndrome is based on gross examination, where the main observation is a thymus severely atrophic and the gastrointestinal tract empty, and the following characteristic histologic lesions: thymic atrophy, superficial lymphoplasmacytic fundic gastritis, villus atrophy in the small intestine, superficial colitis, lymphocytic and neutrophilic rhinitis, and mild nonsuppurative meningoencephalitis. Research on PFTS has explored various factors contributing to the syndrome, including viral agents, genetic predisposition, and nutritional deficiencies. Studies have identified potential infectious agents, but the definitive association with the syndrome remains unclear. Genetic predisposition has also been suggested to play a role during PFTS, identifying potential boars to individually contribute to PFTS and paternity tests have linked affected piglets to certain boars, suggesting individual susceptibility. In this review, we will explore the contributing factors which may be involved in the development of the syndrome, as well as examine the current knowledge on its diagnosis and pathogenesis.

**Conclusion:**

PFTS presents a significant challenge in the swine industry due the unknown aetiology and the difficulty to establish an appropriate diagnosis of the syndrome. Therefore, additional research is needed to investigate the microbial, genetic, and environmental factors that influence PFTS, as this is crucial for developing targeted control measures and potential treatments.

## Introduction

The history of swine production has been marked by the emergence of several syndromes that initially lacked a clear aetiology, that was Porcine Reproductive and Respiratory Syndrome (PRRS), firstly known as mystery swine disease or blue ear pig disease [1], Porcine Postweaning Multisystemic Wasting Syndrome (PMWS) [2, 3], or Porcine Dermatitis and Nephropathy Syndrome (PDNS) [4]. The isolation and identification of the aetiological agent of these syndromes have contributed to elucidate their pathogenesis and more importantly to their control.

Far from considering that the emergence of new diseases had ended, in 2008 a new syndrome, characterised by outbreaks of postweaning “starve-outs”, affecting up to 15% of nursery pigs, appeared. In North America, several farms reported an increase in the number of piglets showing anorexia, lethargy, progressive weakening, and death, mainly within the first weeks after weaning [5]. This syndrome was initially named as “postweaning catabolic syndrome” [5], “postweaning wasting/catabolic syndrome” [6], “postweaning fading pig-anorexia syndrome” [7] and “failure to thrive syndrome” [8]. In 2010, the International Pig Veterinary Society Congress (IPVS) in Vancouver (Canada) agreed to name this condition as “Periweaning Failure to Thrive Syndrome” (PFTS) because of the following reasons: the age of onset corresponded to weaning, it should not be confused with PMWS, and the origin or aetiology may begin during the suckling phase. It was better to use the term “periweaning” instead of “postweaning” because the management or infectious factors both pre- and postweaning could dispose to the development or risk of PFTS [9]. In this regard, it is also important to highlight that weaning is a critical time point for piglets due to the transition from liquid to solid food, and some piglets could fail to adapt and die from starvation [10].

Although the aetiology of PFTS is currently unknown, it is thought that it may be caused by the conjunction of several predisposing factors: infectious and non-infectious (such as management, environmental or nutritional). However, until now, the scientific community is not able to link the clinical and pathological presentation of this syndrome with specific causes [11].

## Impact in the swine production

Initially, these “starve-outs” were reported at a low prevalence, affecting up to 0.5% of total weaned pigs, and were, therefore, associated with minimal economic losses. Nevertheless, the importance of the disease has raised over time [5] due to the increasing number of PFTS cases reported in pig farms over the past few years worldwide [12]. Thus, this syndrome has been recognised in several North American farms [5] and later in Spain [13] and Brazil [14]. Although there are no clear records in the different countries affected by the disease, an estimated average prevalence of 4.3% has been reported in the United States and Canada [12] with a morbidity as high as 20% and high mortality (9). In the case of Spain, where PFTS was diagnosed for the first time in 2012 [13], a total mortality of 4.9% was recorded in the transition phase, which was also associated with stunted growth of pigs [15]. One of the main challenges in controlling this syndrome is the complexity of achieving an assertive diagnosis to differentiate it from other diseases [11], reason why the status of PFTS in the countries where it was initially described remains unknown [13].

## How to recognise and diagnose PFTS?

**Fig. 1:**
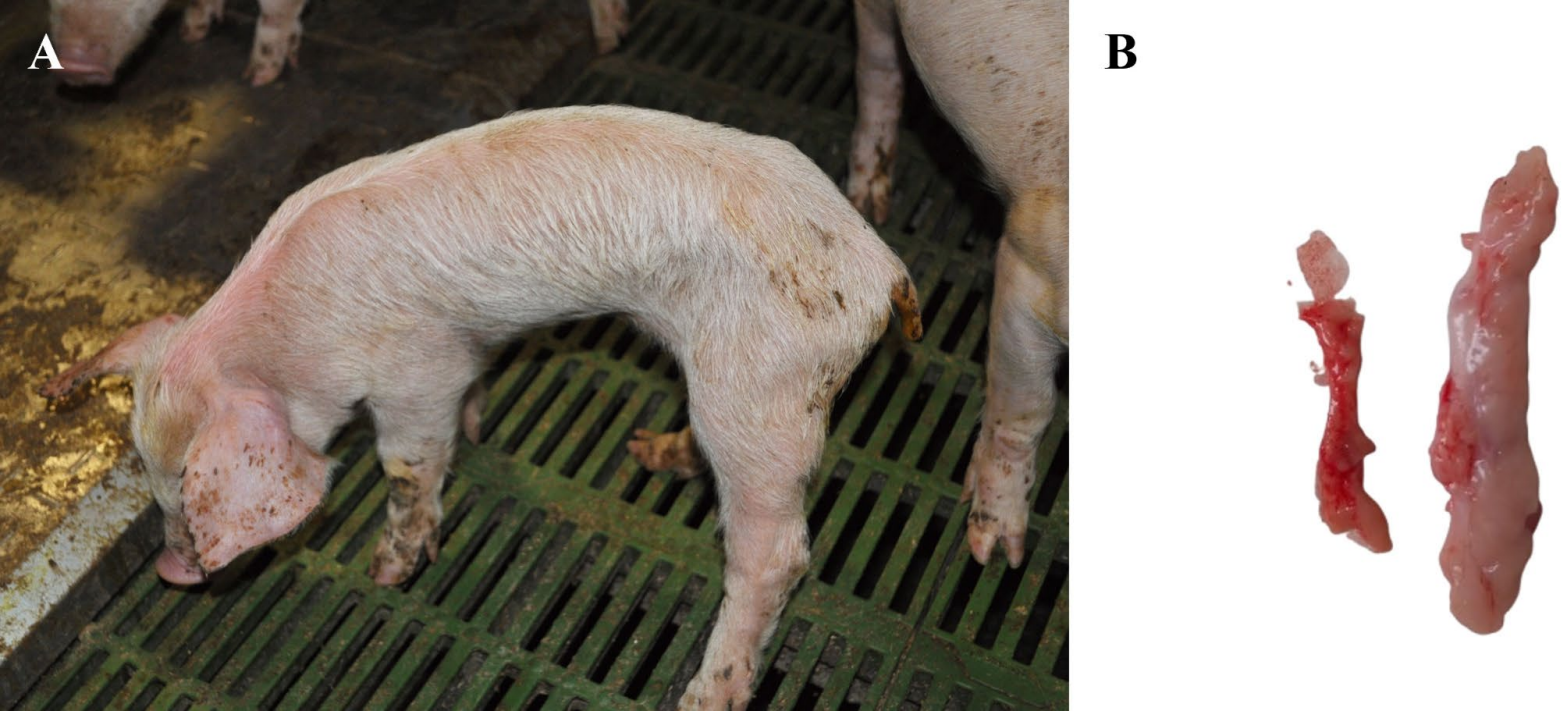
Macroscopic lesions observed in piglets affected with PFTS. Affected-piglet showing marked muscle weakness and loss of body condition (**A**). Cervical thymic atrophy, in the left, in comparison with a cervical thymus of a healthy pig, in the right. (**B**)

A proposed case definition for this syndrome may include the progressive debilitation of weaned pigs in the absence of discernible and detrimental infectious, nutritional, management or environmental factors that can explain the clinical syndrome. At weaning, affected pigs have an average or above average body weight, and neither affected pigs nor their cohorts show signs of residual disease from the suckling phase. Within 7 days of weaning, affected pigs are anorexic, lethargic and showing hollow abdomen. Lack of growth is noticeable in the second week after weaning and usually they can also be seen lying all together and forming piles as an unspecific sign as if they were chilled. In the third week after weaning, there is a rapid deterioration, showing marked muscle weakness and loss of body condition **(**Fig. 1A**)**. Diarrhoea could be also present, although it is not a consistent clinical sign and it is usually seen only in few animals. Some PFTS-sick piglets on all affected farms show repetitive oral behaviour such as licking, chewing, or chomping. In affected farms, batch morbidity and mortality vary over time, but lethality is high [9, 11, 16].

At necropsy, PFTS-affected pigs show scant ingesta in the stomach, and the small intestine and colon are frequently empty or with pasty to liquid content [9, 11, 17]. In early stages of the disease there is a mild thymic atrophy, progressing to bilateral atrophy of the cervical **(**Fig. 1B**)** and also thoracic thymus in late stages [11, 17, 18]. Another unspecific lesion that can be observed in affected animals is a mild to severe cranioventral consolidation of the lungs compatible with a process of suppurative bronchopneumonia [11, 17].

**Fig. 2:**
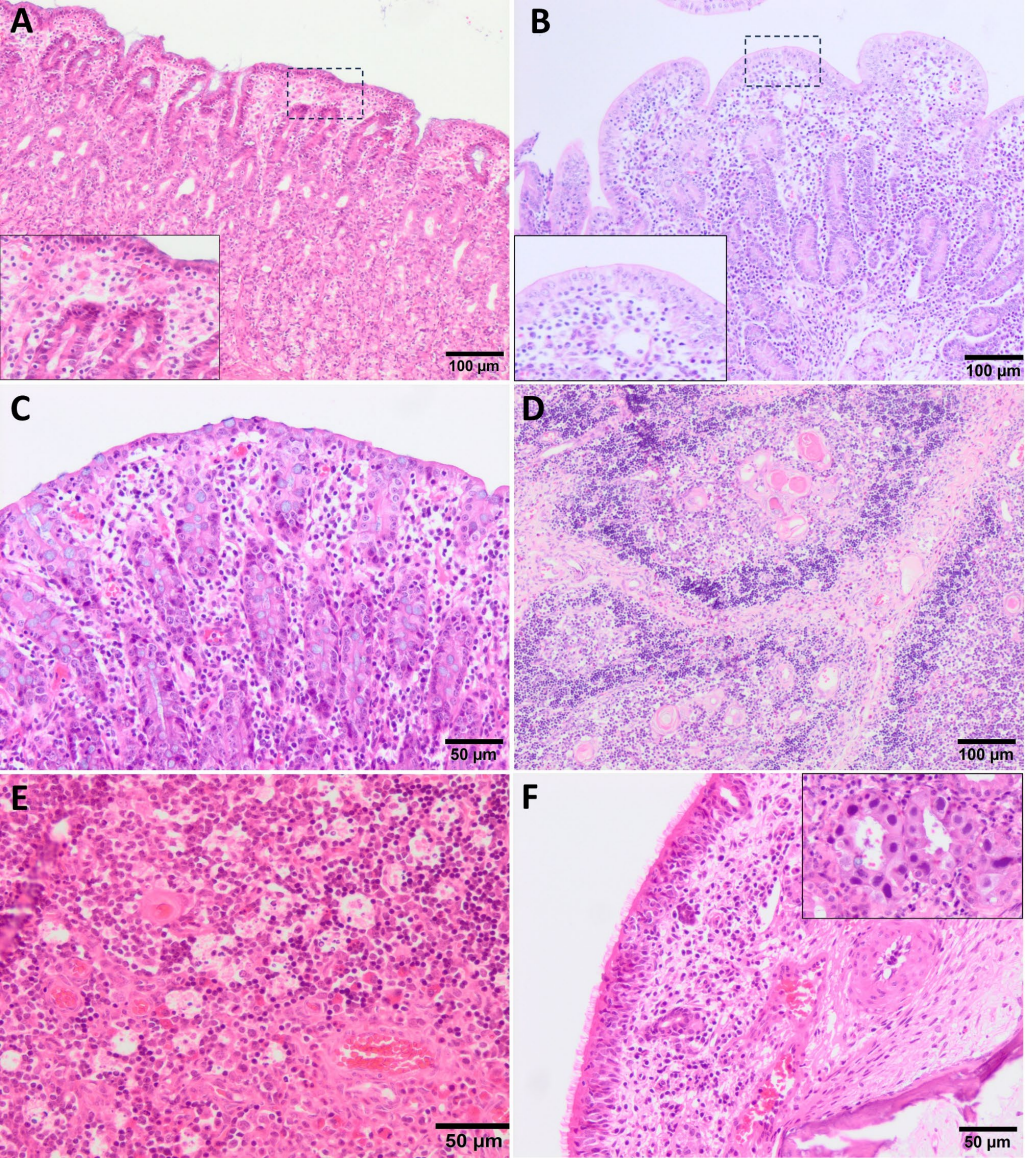
Histologic lesions in piglets with periweaning failure-to-thrive syndrome. H&E. A mild to moderate subepithelial lymphocytic infiltrate in the lamina propria of the gastric fundus and the superficial foveolar cells lack or have reduced cytoplasmic mucus (**A**). Small intestine with villus atrophy and lower villi/crypt (V/C) ratio (**B**). Colon with a superficial lymphocytic infiltrate (**C**). Thymic atrophy with a severe depletion of the lymphoid cells that constitute the cortex of the organ (**D**). Numerous tingible body macrophages and apoptotic bodies giving the appearance of a ‘starry sky’ to the cortex of the thymus (**E**). Mild leucocytic infiltration in the lamina propria of the nasal mucosa and inclusions bodies caused by porcine cytomegalovirus (PCMV) (**F**)

**Fig. 3:**
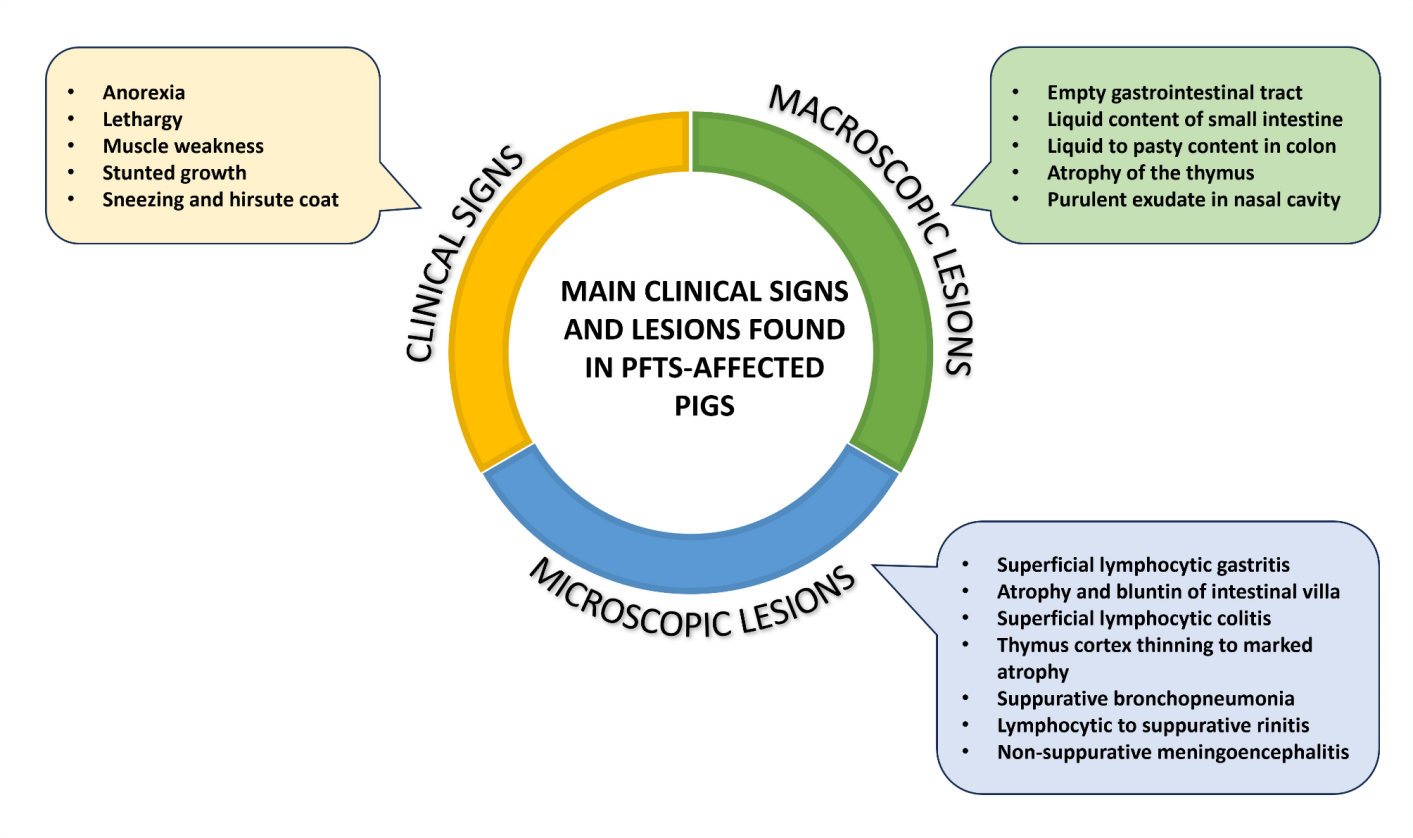
Main clinical signs and lesions found in PFTS-affected pigs

Although PFTS has a non-specific histopathological picture, there are several characteristic microscopic lesions that may be helpful in the diagnosis. A mild to moderate subepithelial lymphocytic infiltrate is observed in the lamina propria of the gastric fundus and the superficial foveolar cells of the affected fundus lack or have reduced cytoplasmic mucus appearing as a simple cubic epithelium without the accumulation of mucinous material in the cytoplasm, and are attenuated, low cuboidal, squamous, or multifocally absent [17] **(**Fig. 2A**)**. In the small intestine (duodenum, jejunum and ileum) villus atrophy and lower villi/crypt (V/C) ratio are observed [17] **(**Fig. 2B**)**, accompanied occasionally by a mild to moderate lymphocytic infiltrate in lamina propria. In colon a superficial lymphocytic infiltrate is also remarkable **(**Fig. 2C**)**. The thymic atrophy is characterised by severe depletion of the lymphoid cells that constitute the cortex of the organ, giving rise to a lower ratio cortex/medulla (C/M) **(**Fig. 2D**)**, which in health animals is around 2:1. Numerous tingible body macrophages and apoptotic bodies are also noticeable, giving the appearance of a ‘starry sky’ to the cortex **(**Fig. 2E**)**. In this regard, animals with PFTS often have such an intense reduction of the cortex that it is indistinguishable from the medulla of the organ. Thymic (Hassall’s) corpuscles show varying degree of neutrophil infiltration, can range from a small number of infiltrating cells between the reticular epithelial cells to the entire corpuscle being filled with inflammatory cells [19]. Also, perivascular lymphocytic cuffs have been occasionally found in the brain and meninges, although they are not observed in a particular area of the brain. It is important to mention that a high percentage of piglets exhibits infiltrate in the nasal mucosa constituted from neutrophils to lymphocytes **(**Fig. 2F**)**, and occasionally associated, but not always, with the presence of intranuclear basophilic inclusions bodies caused by porcine cytomegalovirus (PCMV, also called *Suid herpesvirus 2*, SHV2) **(**Fig. 2F, inset). Moderate to severe, case-dependent, bronchopneumonia has been also observed in PFTS-sick pigs (9, 11, 17, 20), lesions that could not be directly related to the disease but with the immunosuppressed status of the animals. The Fig. 3 summarises the main clinical, macroscopic, and histopathological changes observed in PFTS-affected piglets.

The pathogenesis of these lesions is currently unclear, but above-mentioned lesions are highly prevalent in affected piglets and serve as pathological hallmarks of PFTS. Moeser et al. [21] have shown alterations in intestinal barrier function, associated with increased intestinal permeability, electrogenic ion transport, and morphology of intestinal villus and epithelium. Notably, PFTS pigs displayed intestinal dysfunction and morphological abnormalities independent of anorexia. Weaning-associated stress is known to impact intestinal health, but PFTS pigs showed more severe and persistent intestinal damage compared to controls. Despite sharing stress factors with healthy weaned pigs, PFTS pigs exhibited more pronounced barrier dysfunction, suggesting that a combination of post-weaning (PW) factors, rather than a specific infectious agent or stress alone, might contribute to the syndrome. Additionally, although PFTS pigs did not show typical signs of inflammation, they had distinctive electrogenic ion transport anomalies and fluid accumulation in the intestines. The study also found that PFTS pigs failed to restore normal villus height and crypt proliferation during the PW period, unlike control pigs, indicating an impaired ability to recover from intestinal injury (reduced villus height and crypt hyperplasia). The jejunum and ileum of pigs affected by PFTS show significant changes in short-circuit current (Isc), which is a measure of net electrogenic ion transport. Specifically, the jejunum exhibits a marked negative Isc on days 4 and 11 PW, indicating a disruption in normal ion transport mechanisms. Conversely, the ileum shows a positive Isc on day 4 PW, which shifts to a large negative Isc by day 11 PW. Control pigs have higher Isc values compared to unweaned pigs, suggesting that PFTS leads to decreased efficiency in electrogenic ion transport. The anomalies in Isc are linked to increased intestinal permeability and disturbances in barrier function. This suggests that PFTS may compromise the intestine’s ability to regulate ion transport effectively, contributing to symptoms such as diarrhoea and other digestive disorders. Control pigs have higher Isc values compared to unweaned pigs, suggesting that PFTS leads to decreased efficiency in electrogenic ion transport. The negative Isc values observed in PFTS pigs imply potential impairments in ion absorption or increased ion secretion. The epithelial morphology of PFTS pigs revealed immature secretory cells, which may contribute to increased permeability and compromised barrier function. These findings suggest that PFTS involves a unique intestinal pathology, distinct from known models of intestinal injury or infection, highlighting the need for further investigation into potential metabolic or other non-infectious causes.

In a pilot study conducted by our research group [18], a severe lymphopenia was observed in PFTS-affected animals in comparison with non-affected animals. Thymuses from affected animals showed a high degree of thymic atrophy, which may point to the death of T-cell precursors and their depletion in blood circulation. However, the cell death markers evaluated (TUNEL, iNOS and cleaved caspase-3) by immunohistochemistry in the thymus from these animals were not particularly overexpressed. Thus, according to our results, mechanisms other than apoptosis involved in depopulation of the thymic cortex could be related to the atrophic process occurring in the thymus of PFTS-affected animals. Likewise, necrosis can be ruled out among the potential mechanism, as necrosis generally is accompanied by an inflammatory response which was not observed in our study [22] and by cellular alterations recognizable in the histopathology (cellular debris, karyolysis, karyorrhexis…).

## Differential diagnosis of PFTS: putative causative agents

Since clinical signs presented in PFTS-affected animals are shared with other porcine diseases, a differential diagnosis including porcine circovirus associated diseases (PCVAD) or PRRS, among others, should be carried out (Table 1). This aetiological differential diagnosis becomes particularly relevant when cachectic and weakened pigs appear shortly after weaning, which are PRRS virus and swine influenza virus negative and vaccinated to PCV2, because it suggests that PFTS has a different clinical entity [9].

**Table 1:** Differential diagnosis of the diseases produced by following pathogens compared to PFTS, based on clinical signs and pathological changes

Pathogen	In common with PFTS	Differential with PFTS
PCMV	Catarrhal rhinitis	No thymic atrophy, enteritis or colitis
PCV2	Debilitation, loss of condition Thymic atrophy	Age of onset: 10–12 weeks of age
PRRSV	Debilitation, loss of condition Thymic atrophy (mainly with high virulent strains)	No enteritis or colitis
Pathogenic *E. coli*	Enteritis and colitis	No thymic atrophy

PCMV, porcine cytomegalovirus; PCV2, porcine circovirus type 2; PRRSV, porcine reproductive and respiratory syndrome virus.

Thymic atrophy is highly suggestive of PFTS, although this lesion is also present in other viral infections such as virulent PRRS-1, like SU1-bel or LENA strains, and PRRS-2, like HuN4 strain, classical swine fever, and PCV2 infections [6]. Noteworthy, previous studies were not able to detect these viruses in PFTS-affected animals, however, it highlights the importance of performing ancillary tests accompanying the pathological diagnosis.

According to Huang and Harding [11], the diagnosis of PFTS requires piglets with [1] compatible clinical signs [2], showing at least two of the three most commonly observed histological lesions, such as thymic atrophy, small intestinal villous atrophy and/or superficial lymphocytic gastritis and, in addition [3], all other relevant porcine diseases and pathogens that may affect young pigs in transition phase and cause a similar clinical presentation must be ruled out.

Although the aetiology of PFTS is still unknown, the implication of different aetiological agents has been suggested (Table 2). In early descriptions, S. Henry, in a personal communication cited by Huang. (2013), considered that PCMV might be responsible for the sneezing and suppurative rhinitis frequently encountered in PFTS pigs. Later, intestinal lesions were associated with porcine enteric calicivirus (PECV) infection, due to its identification by electron microscopy in faeces from some affected pigs, which might be responsible for the small intestine villous atrophy and diarrhoea [6]. On the other hand, haemagglutinating encephalomyelitis virus (HEV), a group 2 porcine coronavirus that causes vomiting and wasting disease, was detected in some PFTS pigs, but data showing association between HEV and PFTS was not presented [17]. In another study using metagenomics, an increased number of DNA copies of ungulate bocaparvovirus 2 (BoPV2), ungulate protoparvovirus 1 (PPV1) and porcine circovirus type 3 (PCV3) was identified in PFTS-affected pigs compared to healthy animals [24]; however, this study was carried out in organs such as lung, kidney and liver, not including target organs for PFTS diagnosis, such as the thymus, stomach or intestine, essential organs to confirm the disease. Although PCV3 DNA has been identified in five out of ten selected PFTS cases associated with periarteritis [25], its association with the syndrome and lesions has not been demonstrated to date. The enteric virome from rectal swabs of healthy and wasting piglets from birth to 9 weeks of age concluded that the dynamics of rotaviruses and astroviruses sharply increase one week after weaning in both groups, discarding the role of these pathogens in PFTS [26]. As above mentioned, there are still no studies which directly link the lesions observed in PFTS-affected animals with a specific pathogen involved in the onset of such lesions.

In order to rule out an infectious cause as the origin of PFTS, studies have been carried out to test the presence of different pathogens in organs from PFTS-affected animals. Results from these investigations are shown in Table 2 (modified from Huang et al.) [17].

**Table 2:** Determination of different pathogens in cases of PFTS

Pathogens	Results	Reference
Astrovirus	Positive by PCR and NGS in rectal swab samples	[26]
HEV (Betacoronavirus 1, BCoV-1)	Positive by PCR in tonsil	[17]
	Negative by PCR in lung, kidney, small and large intestine and brain	
Influenza A virus (H3N2 and H1N1 strains)	Negative by PCR in lung	[17]
PCV2	Positive by PCR in tonsil	[17]
	Negative by IHC in lymphoid organs, stomach, small and large intestine	
PCV3	Positive by PCR in lymphoid tissues, lung, heart, kidney, liver, digestive tract (stomach and/or intestine) and central nervous system	[25]
	Positive by NGS in lung, intestine, kidney, liver and brain	[24]
PCMV (*Suid herpesvirus 2*, SuHV-2)	Positive by PCR in tonsil, lung, kidney, small and large intestine	[17]
PECV	Positive and negative by PCR in large intestine	[17]
PRRSV	Negative by PCR in lung	[17]
Rotavirus A	Negative by IHC in stomach, small and large intestine	[17]
Rotavirus A, B, C, E and H	Positive by PCR and NGS in rectal swabs	[26]
TGEV	Negative by IHC in stomach, small and large intestine	[17]
*Torque teno virus 1 and 2 (*TTV-1, 2)	Negative by PCR in spleen or bone marrow	[17]
*Brachyspira hyodysenteriae*	Negative by PCR in large intestine	[17]
*Brachyspira pilosicoli*	Negative by PCR in large intestine	[17]
*Escherichia coli* virulence factors	Negative by PCR in small and large intestine	[17]
*Ungulate bocaparvovirus 2*	Positive by NGS in intestine	[23]
*Ungulate protoparvovirus 1*	Positive by NGS in lung, intestine, kidney, liver, and brain	[24]

HEV, Haemagglutinating encephalomyelitis virus (betacoronavirus 1); PCV2, porcine circovirus type 2; PCV3, porcine circovirus type 3; PCMV, porcine cytomegalovirus; PECV, porcine enteric calicivirus; PRRSV, porcine reproductive and respiratory syndrome virus; TGEV, transmissible gastroenteritis virus (Alphacoronavirus 1); PCR, polymerase chain reaction; NGS, next generation sequencing; IHC, immunohistochemistry.

Finally, some authors tried to reproduce PFTS experimentally. For this purpose, tissue homogenates of tonsil, brain, lung, spleen, stomach, small and large intestines from PFTS-affected pigs which had been resulted positive for PCMV, porcine enteroviruses 1, 2 and 3 and *Helicobacter heilmannii* like organisms were inoculated to snatched-farrowed porcine-colostrum-deprived (SF-pCD) piglets to reproduce the syndrome. The results showed repetitive oral behaviour in both control and inoculated piglets, without alterations in growth or lesions, therefore, the disease could not be reproduced and suggested that the aetiology of PFTS might not be related to the involvement of these agents, although the authors questioned whether the inoculum load might have influenced the outcome [27].

## Is PFTS a non-infectious disease?

Some authors have investigated if PFTS could have a non-infectious origin. In this sense, management causes have been enumerated. These included vaccination, environmental factors, such as assessment of the temperature and ventilation of farms, or dietary causes, since revising the diet of the animals had no cause-effect association [20]. Although vitamin D deficiency was initially identified as a cause of PFTS, it was later discovered that many pig farms in the USA had this problem and it was not associated with PFTS [28].

Different interventions (nutritional, environmental and management interventions) have been tested but none of them were effective. In 2008, two separate groups showed unusual health issues among pigs after weaning, resembling what they termed as “postweaning starve-outs”. These outbreaks affected around 15% of nursery pigs, leading to higher mortality rates on affected farms compared to usual rates [6]. The syndrome’s occurrence was erratic, making it hard to predict. In a farm in western Canada dealing with this issue, various interventions like adjusting ventilation, feed, water sources, administering antibiotics, modifying water nipples, and using electrolytes failed to address the problem [5]. However, drying out the farrowing and nursery rooms with aerosolised hydrated lime seemed to be somewhat effective in reducing mortality, though the evidence supporting its effectiveness was not conclusive [23]. Nevertheless, this partial success hinted at the possibility of an infectious agent contributing to these outbreaks.

A genetic predisposition to PFTS has been also suggested [29–31]. In this way, a paternity test in two Spanish and one Polish farm was able to identify that most of the PFTS-affected piglets were linked to one or two specific boars, which might also indicate an individual susceptibility [30]. This hypothesis was supported by the dramatic decrease in the prevalence of PFTS on-farm after the removal of the “problematic” boars; while the prevalence of PFTS-affected pigs remained the same in farms without changes.

Additionally, in order to determinate if PFTS have a genetic aetiology, Ramis et al. [32] performed a GWAS (Genome-wide association study) in 48 piglets using Illumina’s Porcine 60 K chip, and found an association between the SNPs ASGA0019060, ALGA0024126, H3G10012922 (SSC9) and MARC0043456 (SSC14), but the relationship was not significant, possibly due to the low number of animals analysed in the study so it would be necessary to increase the number of affected piglets to find any SNP associated with the syndrome. Another study tried to link genetic markers and the onset of the pathology in a crossbred swine population from Southern Brazil, finding four chromosomal regions associated with PFTS predisposition, one located in SSCX, one in SSC8 and the other two in SSC14 regions [14]. Notably, these regions harboured crucial candidate genes associated with behavioural changes in humans, particularly those related to depression. This suggests that the health condition may be linked to neurological disorders, affecting vulnerable piglets when exposed to stressful events like the weaning process [14]. Finally, Bertolini et al. [33] examined 119 piglets using the GeneSeek Genomic Profiler 80 K SNP chip, finding 17 regions potentially related to PFTS predisposition. The regions were detected in SSC3, SSC6 and SSC11, which showed moderate divergence between affected and control animals, particularly three haplotypes in affected animals and controls in SSC6 and SSC11. These regions contained genes that have been previously linked to phenotypes associated with the syndrome such as depression and atrophy of the intestinal villi. However, the authors believe that further genomic research is needed to clarify the aetiology and pathogenesis of PFTS. More research in the genetics and genomics is needed to study concrete phenotypes that can be related to the predisposition in pigs to develop the syndrome.

## Conclusions and future perspectives

At present, it is still very difficult to establish both aetiology and diagnosis of PFTS. It remains as a complex and poorly understood condition in swine, marked by significant growth retardation and non-specific clinical signs. Although reported in various countries, including the United States, Canada, Spain, and Brazil, its prevalence and impact remain unclear due to difficulties in diagnosis and distinguishing it from other diseases. The multifactorial nature of PFTS, involving both potential infectious and non-infectious factors, further complicates the development of effective management strategies. Ongoing research is critical to unravel the underlying mechanisms, enhance diagnostic precision, and develop targeted interventions to reduce its impact on the swine industry.

## Data Availability

No datasets were generated or analysed during the current study.

## Abbreviations

BoPV2: Bocaparvovirus 2
GWAS: Genome-Wide Association Study
HEV: Haemagglutinating Encephalomyelitis Virus
IHC: Immunohistochemistry
IPVS: International Pig Veterinary Society Congress
NGS: Next Generation Sequencing
PCV2: Porcine Circovirus type 2
PCV3: Porcine Circovirus type 3
PCVAD: Porcine Circovirus Associated Diseases
PCMV: Porcine Cytomegalovirus
PCR: Polymerase Chain Reaction
PDNS: Porcine Dermatitis and Nephropathy Syndrome
PECV: Porcine Enteric Calcivirus
PFTS: Periweaning Failure to Thrive Syndrome
PMWS: Porcine postweaning Multisystemic Wasting Syndrome
PPV1: Ungulate Prrotoparvovirus 1
PRRS: Porcine Reproductive and Respiratory Syndrome
PRRSV: Porcine Reproductive and Respiratory Syndrome virus
SHV2: Suid Herpes Virus 2
TGEV: Transmissible Gastroenteritis Virus (Alphacoronavirus 1)

## References

[1] OIE. Office International des Épizooties. World Animal Health 1991. Animal health status and disease control methods (Part one; Reports). 1992;7(2):126.

[2] Tischer I, Rasch R, Tochtermann G. Characterization of papovavirus-and picornavirus-like particles in permanente pig kidney cell lines. Zentralbl Bakteriol (Orig A). 1974;226(2):153–67. 4151202

[3] Tischer I, Gelderblom H, Vettermann W, Koch MA. A very small Porcine virus with circular single-stranded DNA. Nature. 1982;295(5844):64–6. 7057875 10.1038/295064a0

[4] Smith WJ, Thompson JR, Done S. Dermatitis/nephropathy syndrome in pigs. Vet Rec. 1993;132:47. 8442343 10.1136/vr.132.2.47-b

[5] Dufresne L, Fangman TJ, Henry S. Post-weaning catabolic syndrome–complexities and perspectives. Proceedings of the Allen D. Leman Swine Conference, St. Paul, June 10 to 12, 2008.

[6] Gauvreau H, Harding J. Why are these nursery pigs dying? An ongoing field investigation into a farm with elevated nursery mortality associated with non-PRRS/PCV2 post weaning starvation. SK: In: Proc West Can Assoc Swine Vet Conf. Saskatoon; 2008.

[7] Rossow K. Postweaning fading pig/anorexia syndrome. National Hog Farmer. 2010. Available from: http://nationalhogfarmer.com/weekly-preview/0628-postweaning-fading-pig-anorexia/

[8] Vansickle J. Researchers scramble to solve failure to thrive syndrome. *National Hog Farmer.* 2008. Available from: http://nationalhogfarmer.com/health-diseases/0915-researchers-trying-solve-syndrome

[9] Henry YS, Friendship R, Schwartz K, Harding J. Clinical presentation, case definition, and diagnostic guidelines for porcine periweaning failure to thrive syndrome. J Swine Health Prod. 2011;19(6):340–344.

[10] Arnaud EA, Gardiner GE, Lawlor PG. Selected nutrition and management strategies in suckling pigs to improve post-weaning outcomes. Animals. 2023;13:1998. 37370508 10.3390/ani13121998 PMC10294848

[11] Huang Y, Harding JC. Pathological features and proposed diagnostic criteria of Porcine periweaning failure-to-thrive syndrome. Vet Pathol. 2015a;52(3):489–96. 10.1177/0300985814542810. 25051955 10.1177/0300985814542810

[12] O’Sullivan TL, Harding JCS, Friendship R, Henry S, Madson D, Schwartz K. Estimated prevalence and impact of periweaning failure to thrive syndrome in Canada and the united States. J Swine Health Prod. 2014;22(1):24–8.

[13] Segalés J, Martínez J, Vidal E, Kekarainen T, Bragulat J, Quintilla C, et al. Periweaning failure to thrive in pigs in Spain. Vet Rec. 2012;170(19):499. 10.1136/vr.e3301. 22589037 10.1136/vr.e3301

[14] Zanella R, Morés N, Morés MA, Peixoto JO, Zanella EL, Ciacci-Zanella J, et al. Genome-wide association study of periweaning failure-to-thrive syndrome (PFTS) in pigs. Vet Rec. 2016;178(26):653. 10.1136/vr.103546. 27162284 10.1136/vr.103546

[15] SIP Consultors. Informe sobre datos de producción en España en 2020. 2021. Available from: http://www.sipconsultors.com/

[16] Friendship B, Harding J, Henry S. Periweaning failure to thrive syndrome (PFTS) – difficulties of investigating an emerging clinical problem. Proc Allen D. Leman Swine Conf. St Paul, Minnesota. 2010:73–78.

[17] Huang Y, Gauvreau H, Harding J. Diagnostic investigation of Porcine periweaning failure-to-thrive syndrome: lack of compelling evidence linking to common Porcine pathogens. J Vet Diagn Invest. 2012;24(1):96–106. Epub 2011 Dec 6. PMID: 22362939. 22362939 10.1177/1040638711425939

[18] Muñoz-Hidalgo A. Alteraciones tímicas y sanguíneas en lechones afectados por el síndrome del fallo del desarrollo peridestete (PFTS) [Undergraduate thesis]. Córdoba: Facultad de Veterinaria, Universidad de Córdoba; 2021.

[19] Pallarés FJ, Gómez S. Unusual histopathologic features in thymic corpuscles associated with Porcine periweaning failure-to-thrive syndrome. J Vet Diagn Invest. 2019;31(4):601–3. 10.1177/1040638719846208 31006384 10.1177/1040638719846208 PMC6857017

[20] Pallarés FJ. Síndrome Del Fallo de desarrollo Peridestete (PFTS). Info Ingaso. 2013;11:7–10.

[21] Moeser AJ, Borst LB, Overman BL, Pittman JS. Defects in small intestinal epithelial barrier function and morphology associated with peri-weaning failure to thrive syndrome (PFTS) in swine. Res Vet Sci. 2012;93(2):975–82. 10.1016/j.rvsc.2012.01.003. 22284769 10.1016/j.rvsc.2012.01.003

[22] Jordán J. (2003). Apoptosis: muerte celular programada. Offarm. 2023;22(6), 100-6.

[23] Huang Y. December. Is porcine periweaning failure-to-thrive syndrome an infectious disease? [dissertation]. Saskatoon: University of Saskatchewan; 2013. Available from: https://core.ac.uk/download/pdf/226122438.pdf

[24] Franzo G, Kekarainen T, Llorens A, Correa-Fiz F, Segalés J. Exploratory metagenomic analyses of periweaning failure-to-thrive syndrome-affected pigs. Vet Rec. 2019;184(1):25. 10.1136/vr.105125. 30413677 10.1136/vr.105125

[25] Cobos À, Sibila M, Alomar J, Pérez M, Huerta E, Segalés J. Retrospective assessment of Porcine circovirus 3 (PCV-3) in formalin-fixed, paraffin-embedded tissues from pigs affected by different clinical-pathological conditions. Porc Health Manag. 2022;8:51. 10.1186/s40813-022-00293-8. 10.1186/s40813-022-00293-8 PMC9720923 36471405

[26] Folgueiras-González A, van denh Braak R, Deijs M, Kuller W, Sietsma S, Thuring V, et al. Dynamics of the enteric Virome in a swine herd affected by Non-PCV2/PRRSV postweaning wasting syndrome. Viruses. 2021;13:2538. 10.3390/v13122538. 34960807 10.3390/v13122538 PMC8705478

[27] Huang Y, Harding JC. Attempted experimental reproduction of Porcine periweaning failure-to-thrive syndrome using tissue homogenates. PLoS ONE. 2014;9:e90065–5. 24594806 10.1371/journal.pone.0090065 PMC3940845

[28] Vansickle J. Fixing vitamin D deficiency. *National Hog Farmer.* 2012. Available from: https://www.nationalhogfarmer.com/hog-nutrition/fixing-vitamin-d-deficiency

[29] Huang Y, Harding JCS. Do genetics play a role in Porcine periweaning failure-to-thrive syndrome? Vet Rec. 2015b;176(23):594. 10.1136/vr.h2899. 26044686 10.1136/vr.h2899

[30] Ramis G, Marco E, Magaña V, González-Contreras P, Swierczynski G, et al. Evidence that periweaning failure-to-thrive syndrome (PFTS) has a genetic predisposition. Vet Rec. 2015;176:596. 25820322 10.1136/vr.102748

[31] What are genome-wide association studies?. *MedlinePlus Genetics*. Available from: https://medlineplus.gov/genetics/understanding/genomicresearch/gwastudies/ Accessed 22 March 2022.

[32] Ramis G. Genome wide-association study in animals affected by periweaning fail-to-thrive syndrome (PFTS): preliminary results. 24th International Pig Veterinary Society Congress, 8th European Symposium of Porcine Health Management; 2016; Royal Dublin Society, Dublin, Ireland.

[33] Bertolini F, Yang T, Huang Y, Harding JCS, Plastow G, Rothschild M. Genomic investigation of Porcine periweaning failure to thrive syndrome (PFTS). Vet Rec 2018:Vetrec-2017-104825. 10.1136/vr.104825 29695451

